# How two word-trained dogs integrate pointing and naming

**DOI:** 10.1007/s10071-012-0494-x

**Published:** 2012-04-24

**Authors:** Susanne Grassmann, Juliane Kaminski, Michael Tomasello

**Affiliations:** 1 Department of Developmental Psychology, Heymans Institute, University of Groningen, Grote Kruisstraat 2/1, 9712 TS Groningen, The Netherlands; 2 Department of Developmental and Comparative Psychology, Max Planck Institute for Evolutionary Anthropology, Leipzig, Germany

**Keywords:** Domestic dogs, Communicative intentions, Pointing, Word learning

## Abstract

Two word-trained dogs were presented with acts of reference in which a human pointed, named objects, or simultaneously did both. The question was whether these dogs would assume co-reference of pointing and naming and thus pick the pointed-to object. Results show that the dogs did indeed assume co-reference of pointing and naming in order to determine the reference of a spoken word, but they did so only when pointing was not in conflict with their previous word knowledge. When pointing and a spoken word conflicted, the dogs preferentially fetched the object by name. This is not surprising since they are trained to fetch objects by name. However, interestingly, in these conflict conditions, the dogs fetched the named objects only after they had initially approached the pointed-to object. We suggest that this shows that the word-trained dogs interpret pointing as a spatial directive, which they integrate into the fetching game, presumably assuming that pointing is relevant to finding the requested object.

## How two word-trained dogs integrate pointing and naming

Some nonhuman individuals are able to comprehend human symbols in the sense that they respond to them in human-like ways. The best known are individual linguistic apes, who acquire receptive vocabularies of several hundred “words” (e.g., Savage-Rumbaugh et al. 1993). However, chimpanzees (and other great apes) seem not to understand pointing (see Call and Tomasello 2005 for a review). However, recent studies suggest that at least chimpanzees have some sensitivity to pointing and show it when methods used for testing and their environments are changed (Lyn et al. 2010). Furthermore, trained individuals from other species also understand pointing (Herman et al. 1999; Miklósi and Soproni 2006). However, domestic dogs (as a species) seem to be special, in that they seem to be very good at understanding pointing (see Miklósi and Soproni 2006 for a review). Individual domestic dogs have also been trained to comprehend spoken object labels (more than 1000 in one case) (cf., Kaminski et al. 2004; Pilley and Reid 2011). These dogs seem to understand the human use of object labels in some sense referentially, as they fetch designated objects by their labels.

Two of the word-trained dogs (Rico and Chaser) were able to infer referents of novel labels by exclusion. That is, when Rico was asked to fetch *Sirikid* (a novel word for the dog) from a set that comprised several familiar objects and one unknown object, Rico correctly identified the novel object as the referent of the novel label (Kaminski et al. 2004, see also Pilley and Reid 2011). Since word learning by exclusion in human children is considered to rely either on sophisticated inferences about speakers’ communicative intentions (e.g., Clark 1990; Diesendruck and Markson 2001) or on specific word learning mechanisms (Markman 1989; Merriman and Bowman 1989), Kaminski et al.’s (2004) original findings led to a debate as to whether and how dogs’ label repertoire is similar to human vocabularies with respect to these dogs’ understanding of reference. For dogs, Markman and Abelev (2004) suggest that neophilia, a low-level process, rather than understanding of communicative intentions accounts for referent resolution by exclusion.

Some developmental psychologists argue that word-trained dogs’ skills with object labels are very different from a human understanding of reference (Markman and Abelev 2004; Bloom 2004). For example, Bloom (2004) pointed out that for children, object labels are symbols that can be used for a certain kind of object (a category) in a variety of different contexts. For dogs, however, he argues that object labels may be nothing more than highly specific commands to fetch particular objects. In response to that, Pilley and Reid (2011) showed that Chaser, a word-trained Border collie, can perform actions other than fetching with named objects. This suggests that Chaser might indeed understand that labels like “ball” are not specific fetching commands but refer to objects. However, one could argue that Chaser may have merely learned complex action commands (e.g., “take ball” and “nose ball”) during training. That is to say, it is not clear from Pilley and Reid’s study whether Chaser really separated the object label from the action label and thus understands the different functions.

Markman and Abelev (2004) raised another point against the view that word-trained dogs understand object labels referentially: They argue that dogs’ correct identification of referents of words might not reflect the dogs’ understanding of human’s referential intentions, but rather be a conditioned response. They suggest that a demonstration is needed that dogs are able to understand humans’ referential intentions. Such understanding of referential intentions is required in object choice task when study participants have to figure out what a human wants them to fetch. A recent study by Kaminski et al. (2009) suggests that word-trained dogs may be able to read referential intention: It was found that, without previous training, dogs used replicas in order to fetch objects as desired by a human. Thus, these dogs show some understanding of the referential/symbolic function of human communicative signs.

Referential understanding (as developmental psychologists study it) includes understanding of the co-reference of various aspects of a multimodal referring expression, for example, when a mother points to an object for her baby and says, “look, that is a ball.” (cf., Koenig and Echols 2003). In human communication, both pointing and object labels can be used referentially. Human infants understand communicative intentions expressed in pointing gestures even prelinguistically (Behne et al. 2005). Further, in Western cultures, parents often point at objects and name them for their children (Masur 1997).

Importantly, and in sharp contrast to other animals, by the time human children comprehend pointing, they also start producing it for various functions (Liszkowski et al. 2007; Tomasello et al. 2007) and caretakers often respond to children’s pointing by naming the pointed-to objects (Hannan 1992). So it is not surprising that from the outset of word learning, children know and expect that pointing and object labels co-refer and thus express a single coherent referential intention. That is, when an adult points to an object, infants as young as 13 months of age expect that the word she is saying is the object’s name (Gliga and Csibra 2009). This expectation is the core of referential understanding in children.

Interestingly, Grassmann and Tomasello (2010) demonstrated that when a speaker points to one object and simultaneously uses an object label, children assume that the pointed-to object is the object she intended to refer to, even when the spoken word was not the name the child would expect to hear for the pointed-to object. For example, when the adult verbally requested “the car” while pointing to a novel object, young children handed the adult the novel object. On the basis of the children’s object selection in various conflict conditions, Grassmann and Tomasello argue that children do not ignore the spoken object label but integrate the current use of the spoken word with their previous word knowledge. For example, they probably assume that the novel object is a car. Similarly, when the adult pointed at a car and verbally named it “the modi” (a novel word), children inferred that the speaker meant to refer to the car and that “modi” was probably a superordinate term (cf., Liittschwager and Markman 1994; Mervis et al. 1994).

We suggest that investigating word-trained dogs’ interpretation of bimodal referential expressions in which pointing and object labels are used simultaneously would provide a good test case as to whether they understand human referential intentions in a manner similar to humans. The current study therefore asks whether word-trained dogs integrate their understanding of the pointing gesture (which they share with other dogs) with interpreting the reference of spoken words (in the fetching game). In three conditions, we assessed (1) whether word-trained dogs use the pointing gesture in order to disambiguate the reference of a novel word between two novel objects (Ostensive Naming condition), and (2) how word-trained dogs interpret a referential act in which the speaker is using a novel word while pointing to an object for which the dog had learned another label. In other words, would dogs allow multiple names for one object (Novel Label Conflict)? (3) How word-trained dogs respond to a speakers’ referential act when in which a known word is used while pointing to a novel object. In other words, do dogs accept more than one referent per label without extensive associative training (Familiar Label Conflict)?

In order to establish baselines for dogs’ interpretation of pointing, familiar words, and novel words in isolation, three further conditions were conducted: a Pointing Baseline, in which no novel word was used, a Familiar Label Baseline, in which the dogs were simply tested for their vocabulary, and a Mutual Exclusivity condition, in which we assessed reference resolution by exclusion. Since we were interested in dogs’ inferential skills about human referential expressions, retention of novel label-object mappings was not tested.

We hypothesized that if the word-trained dogs were able to fetch objects based on pointing in the Pointing Baseline (a cue the owners do not use in the fetching game) and if they were also able to rely on the pointing gesture in order to disambiguate a speaker’s reference between two novel objects, then this would provide initial evidence for dogs’ sophisticated skills in integrating multimodal communication. This would be a prerequisite to integrating pointing and naming in a similar manner to how human children integrated them in the conflict conditions. In this case, the dogs should fetch the pointed-to object. Alternatively, the dogs may preferably rely on words and not integrate words and gesture to a single interpretation when the two seemingly contradict one another, simply because they have been trained to obey to words in the fetching game.

## Methods

### Participants

Two Border collies (1 male, age 8 year; 1 female, age 9 year) participated in this study. Both dogs were family dogs that lived as pets with their owners and were experienced in fetching objects by their labels. Paddy knew labels for approximately 60 objects, and Betsy knew 300 object labels at the time they were tested. Both dogs play the fetching-objects-by-name game one to 2 h per week with their owners. Betsy is introduced to novel objects and labels on an irregular basis (birthday gifts, gifts brought by visitors). Her word training was done in a ritual manner; first, the owner tells the dog that there will be a new toy. Then, the novel object is presented together with the novel name. Then, the new object is thrown, and the owner uses the novel word in her command to the dog to fetch the object. Paddy receives one or two new objects each month. His word training is similar to Betsy’s: First, the object is presented to the dog, the name is given, then the object is handed to the dog for exploration, then it is hidden or thrown away, and Paddy is asked to fetch it.

### Materials and design

We examined the dogs’ object choice in six conditions: two baseline conditions (Pointing Baseline, Familiar Label Baseline) and four experimental conditions: two conflict conditions (Familiar Label Conflict condition and Novel Label Conflict condition) and two nonconflict conditions (Mutual Exclusivity, Ostensive Naming). The dogs received 12 trials in each of the four experimental conditions. The experimental conditions were presented in three blocks of four to six trials. In each session, the dogs received one to three blocks of different conditions with the order of the blocks counterbalanced across sessions. The Ostensive Naming Condition was assessed 2 years after initial data collection in two and three sessions, respectively.^1^ The Familiar Label Baseline and the Pointing Baseline served as fillers.

For each of the experimental trials, a set of six objects was created. For the Ostensive Naming Condition, the set comprised four familiar and two novel objects. For all other experimental conditions, the sets comprised five familiar and one novel object. The familiar objects were randomly chosen from the dogs’ own toys. The novel objects were pets’ or children’s toys for which the owner confirmed prior to the study that they were novel for their dog. For each of these sets, the dogs were successively asked to fetch three objects (without replacement of selected toys): one request in the Pointing Baseline, one request in the Familiar Label Baseline, and one request in an experimental condition. The order of requests was such that the experimental condition was preceded by either the Pointing Baseline or the Familiar Label Baseline 50 % of the time. In addition, the experimental condition was presented either second or third for each set of toys.

The novel labels used in the Mutual Exclusivity Condition, the Ostensive Naming Condition, and the Novel Label Conflict Condition were suggested by the owner. Each novel object was used only once in the entire study, but familiar objects that served as distractors were allowed to occur more than once.

### Procedure

**Fig. 1:**
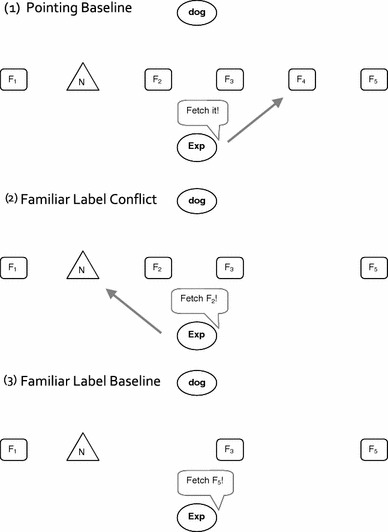
Example of the order of three object requests (one request in each of the two baseline conditions and one request in an experimental condition) for one set of five familiar (F_1_–F_5_) and one novel object (N)

The testing took place in the dog owners’ homes. For each trial, six objects were placed in a row on the floor with 30-cm space between neighboring toys (see Fig. 1). The dog sat approximately 2 m away. Betsy was tested in one room. Her owner held her in the starting position and looked down during the whole trial. For Paddy, two adjacent rooms were used. The objects were placed in one room, and Paddy waited in the doorway to the adjacent room for the request to fetch an object. Then, the experimenter asked the dog to fetch an object—according to condition, different cues were provided. After the dog picked one object, he/she was requested to bring it to the owner (Betsy) or to an assistant in the adjacent room (Paddy). The owner and the assistant gave only neutral responses to the dog’s selection, that is, they never praised the dog irrespective of the dog’s choices. They took the toy from the dog and placed it aside, and the next request started. The dog owners, who were blind to experimental conditions, signaled to the experimenter if they thought that their dog was getting tired. In this case, the session was terminated and continued later in the day or the next day. The position of the novel object was counterbalanced across trials (with the restriction that the novel object was never in the rightmost or leftmost position of the row). After the three requests, all remaining objects were cleared away, and the next set of six objects was placed on the floor.

#### Pointing Baseline

In this condition, the experimenter requested a familiar object by pointing to it (proximal pointing—ca. 10 cm between finger and object). The experimenter always pointed with an extended arm, leaning her body also toward the indicated object. This was necessary in order to make pointing to the objects in the middle of the row of objects identical to pointing to objects placed to the edges of the row of objects. The pointing gesture was accompanied by the verbal request “Fetch it.” The verbal request was repeated twice, and the pointing gesture remained until the dog made a choice. Throughout the trial, the experimenter altered her gaze between the dog and the pointed-to object.

#### Familiar Label Baseline

The experimenter asked the dog to select a familiar object (e.g., “Fetch the car.”). The familiar word was repeated twice. Throughout the trial, the experimenter looked straight to the dog.

#### Ostensive Naming

The experimenter pointed to one of the two novel objects that were positioned next to one another and said, for example, “Fetch the *blicket.*” The novel word was repeated twice.

#### Mutual Exclusivity

The experimenter asked the dog to find the referent of a novel word, for example, “Fetch the *modi.*” The novel word was repeated twice.

#### Familiar Label Conflict

As in the Familiar Label Baseline, the experimenter requested a familiar object by saying its label two times (e.g., “Fetch the *car*.”). Additionally and simultaneously, she pointed to the novel object and alternated her gaze between the dog and the pointed-to object.

#### Novel Label Conflict

This condition is the reverse of the Familiar Label Conflict: The experimenter asked the dog, “Fetch the *toma*” and simultaneously pointed to a familiar object that was positioned next to the novel object. The novel word was repeated twice.

If the dogs did not pick an object, the request was repeated once. Sometimes, a dog fetched the novel object from a set before it was a target. In this case, a new set of six objects was used the whole series of requests repeated (nine times for each dog). This procedure resulted in different numbers of trials. In particular, the number of trials in the experimental conditions varied between 10 and 12, and the number of baseline trials varied between 43 and 54 (see Table 1).

**Table 1:** Number of object retrievals consistent with the label and the pointing gesture in all conditions

	Object selection			
	Labeled object	Pointed-to object	Next to pointed-to object^a^	Other
*Paddy*				
Pointing Baseline (N = 43)		22 (51 %)***	7 (16 %)	14 (33 %)
Familiar Label Baseline (N = 47)	42 (89 %)***			5 (11 %)
Mutual Exclusivity (N = 12)	10 (83 %)***			2 (17 %)
Ostensive Naming (N = 12)		9 (75 %)***	3 (25 %)^b^	
Familiar Label Conflict (N = 10)	7 (70 %)**	2 (20 %)		1 (10 %)
Novel Label Conflict (N = 12)	7 (58 %)**	1 (8 %)		4 (34 %)
*Betsy*				
Pointing Baseline (N = 42) N = 54		19 (35 %)**	6 (11 %)	29 (54 %)
Familiar Label Baseline (N = 46)	43 (94 %)***			3 (6 %)
Mutual Exclusivity (N = 11)	2 (18 %)			9 (82 %)
Ostensive Naming (N = 12)		7 (58 %)**	4 (34 %)^c^	1 (8 %)
Familiar Label Conflict (N = 12)	8 (67 %)**	4 (33 %)		
Novel Label Conflict (N = 11)	6 (55 %)*	3 (27 %)		2 (18 %)

Asterisks indicate performance significantly above chance level. Cells are blank when this option was not available in a condition * *p* < 0.05; ** *p* < 0.01; *** *p* < 0.001

^a^Next to pointed-to object is the labeled object in conflict conditions (except for one case for Paddy in the Novel Label Conflict condition)

^b^2 times the novel object next to the pointed-to novel object, 1 time the familiar object on the other side

^c^3 times the novel object next to the pointed-to object, 1 time the familiar object on the other side

### Coding and reliability

We scored the dogs’ first touch/approach and their object selection (fetching). First touch was coded for the object that the dog first considered by touching it with the nose. Object selection was coded as the object the dog picked up in order to bring it to the owner, the experimenter, or an assistant. A second independent coder coded a randomly selected set of nine trials from each dog from videotape for reliability. The first and the second coding indicated excellent reliability both for first touch (Paddy: Cohen’s Kappa = 0.96, Betsy: Cohen’s Kappa = 0.86) and for selection (Paddy: Cohen’s Kappa = 0.98 Betsy: Cohen’s Kappa = 1).

## Results

For purposes of statistical analyses, the dependent measures were how often the dogs 1) first touched and 2) selected the *pointed*-*to* or the *labeled* object.

### Statistical analysis

The dogs’ behavior was compared to chance level by using a Monte Carlo manipulations (Adams and Anthony 1996; Manly 1997). These simulations were employed in order to simulate random object selection for each condition by controlling for different chance values resulting from not replacing fetched items to sets.^2^ The simulation repeated random object selection for each condition 10,000 times and thereby generated a frequency distribution for simulated (random) number of correct object selection for each condition. The mean of that simulated frequency distribution served as the chance-level value. The *p* value for the comparison of a dog’s object selection against chance in a particular condition was determined as the proportion of simulated object selections in which the number of correct object selection was as high as or higher than that of dog’s actual object selection.

#### Object selection

The dogs’ object selection responses in all conditions are given in Table 1. Statistical analyses comparing the dogs’ object selection against chance level reveal a very good performance with familiar spoken words. Both dogs chose the labeled object above chance level in all conditions in which a familiar label was used. Additionally, Paddy but not Betsy was also able to identify the referent of a novel word in the Novel Label Baseline. Both dogs chose the pointed-to object at above chance levels but nevertheless at a surprisingly low level. A comparison of the two baselines revealed that both dogs chose the requested object more reliably in the Familiar Label Baseline than in the Pointing Baseline (Paddy: χ^*2*^ = 15.95, *df* = 1, *p* < 0.001, Betsy: χ^*2*^ = 35.827, *df* = 1, *p* < 0.001). Therefore, it is not surprising that in the conflict conditions both dogs chose the labeled object at above chance level but chose the pointed-to object only at chance level.

Since most studies employing object choice in order to test dogs’ understanding of pointing use first approach behavior, we ran a second set of analyses based on the dogs’ first touch rather than their fetching.

#### First touch

The dogs’ first touch responses in all conditions are given in Table 2. Paddy approached the labeled object at above chance level in all conditions where a label was available except in the Novel Label Conflict condition. Furthermore, Paddy also approached the pointed-to object at above chance level in all conditions in which a pointing gesture was available. The analysis of Betsy’s first touch behavior revealed that she followed the label at above chance level in all conditions in which a familiar word was used. Furthermore, she approached the pointed-to object at above chance levels when no familiar label was available. A comparison of the two baselines revealed that both dogs approached the requested object *equally* in the Pointing Baseline and in the Familiar Label Baseline. Thus, the dogs’ responses in the conflict conditions indicate that pointing is important in guiding the dogs’ first touch. Nevertheless, the relatively low proportion of correct referent identification in the Pointing Baseline remains surprising. It may be worth noting that both dogs approached or fetched objects that were positioned next to the pointed-to object relatively frequently in the Pointing Baseline (Paddy: 16 % in selection, 9 % in first touch; Betsy: 11 % in selection, 17 % in first touch).

**Table 2:** Number of first approaches consistent with the label and the pointing gesture in all conditions

	First touch			
	Labeled object	Pointed-to object	Next to pointed-to object^a^	Other
*Paddy*				
Pointing Baseline (N = 43)		20 (47 %)***	4 (9 %)	19 (44 %)
Familiar Label Baseline (N = 47)	21 (45 %)***			26 (55 %)
Mutual Exclusivity (N = 12)	6 (50 %)*			6 (50 %)
Ostensive Naming (N = 12)		12 (100 %)***		
Familiar Label Conflict (N = 10)	5 (50 %)*	5 (50 %)*		
Novel Label Conflict (N = 12)	4 (33 %)	8 (67 %)**		
*Betsy*				
Pointing Baseline (N = 54)		32 (59 %)***	9 (17 %)	13 (24 %)
Familiar Label Baseline (N = 46)	26 (57 %)***			20 (43 %)
Mutual Exclusivity (N = 11)	3 (27 %)			8 (73 %)
Ostensive Naming (N = 12)		9 (75 %)***	3 (25 %)^b^	
Familiar Label Conflict (N = 12)	6 (50 %)*	4 (34 %)		2 (18 %)
Novel Label Conflict (N = 11)	2 (18 %)	9 (82 %)*		

Asterisks indicate performance significantly above chance level. Cells are blank when this option was not available in a condition * *p* < 0.05; ** *p* < 0.01; *** *p* < 0.001

^a^Next to pointed-to object is the labeled object in conflict conditions

^b^2 times novel object, 1 time familiar object

#### Comparisons of first touch and selection

For each condition, the dogs’ first touch behavior was compared to their object selection by using McNemar chi-square tests. In the Familiar Label Baseline, both dogs’ response was better at selection than at first touch (both dogs *p* < 0.001). Interestingly, in the Pointing Baseline, Betsy was better at her first approach than at her eventual object selection (*p* < 0.01), while Paddy’s reliance on pointing was equal at first touch and selection.

## Discussion

Both dogs in the current study were skillful in fetching familiar objects requested by an unfamiliar person in a fetching game. Both dogs successfully picked objects indicated by pointing or familiar labels. The dogs also relied on pointing in order to disambiguate the reference of a novel label in the Ostensive Naming Condition. In the conflict conditions, however, when pointing was in conflict with a label, both dogs fetched the labeled object. Interestingly, the dogs did so only after having *first approached* the pointed-to object. One of the dogs (Paddy) was also able to identify the referent of novel object labels by exclusion. Interestingly, the other dog (Betsy) failed to do so. Betsy’s performance in the Mutual Exclusivity condition contradicts claims that neophilia might cause exclusion phenomena in word-trained dogs (cf., Markman and Abelev 2004; Kaulfuß and Mills 2008).

Overall, the dogs’ responses in the conditions where pointing and labeling occurred together (whether in a conflicting manner or in a disambiguating manner) indicate that the dogs attended to pointing and integrated it into the fetching context. This is interesting since the dogs had been trained to fetch objects by name, and thus, arguably, object labels are the most relevant information for the dogs in the fetching game. The importance of object labels is also shown be the fact that both dogs chose the target referent more reliably in the Familiar Label Baseline than in the Pointing Baseline. Despite their training, however, the dogs used pointing in the fetching game, and this may indicate that they understand something about their interlocutor’s referential intentions.

Therefore, the main question for discussion is: Why did the dogs attend to the pointing gesture in the conflict conditions where they could have ignored the pointing? And why did they do it in the way that they did: first approaching the pointed-to object but then *not* fetching it? We suggest that the clue for the answer to that question lies in the fact that in the conflict conditions the named object was always positioned next to the pointed-to object. This made pointing helpful for the dogs in finding the named object among a set of four or five objects. One reason for the dogs following the point could be that movement of the human toward an object is highly salient to dogs and leads them to walk in the indicated direction.^3^ Another reason for the dogs following the pointing gesture might be that the (visual) information that is available to them from 2-m distance is not sufficient to identify the referent of a spoken word. Thus, the dogs’ initial approaches to the pointed-to object might reflect their attempt to gain additional information about where to find the requested object. Especially interesting in this respect is Betsy’s object selection in the Novel Label Conflict: In this condition, Betsy fetched the labeled object (according to exclusion inferences) at above chance level. However, in the Mutual Exclusivity condition, she was not successful with this inference. Thus, it seems as if Betsy needed the speaker’s hint that the requested object is to be found in a certain area of the choice set.

This line of thinking would suggest that the word-trained dogs do integrate the experimenter’s pointing and naming, but they do not understand pointing as indicating a *specific* location or object, but as something a bit looser: a spatial cue that leads them to walk in the indicated direction. Such an understanding would enable them to find a desired object or piece of food in most studies on dogs’ point comprehension. A similar point has been made by researchers suggesting that dogs may interpret pointing as some kind of directive ordering them where to go instead of informing them about/referring to objects in the vicinity (Kaminski 2008; Scheider et al. 2011; Topál et al. 2009; Wobber and Kaminski 2011). Spoken labels, on the other hand, are directly mapped to individual objects through the dogs’ word training. Thus, pointing and words work differently for word-trained dogs. Note that for humans, however, pointing and words are both referential, that is, object-related (see Topál et al. 2009 for a discussion of a similar point how human communicative signals differ for dogs and children).

The fact that the dogs’ object choice in the conflict conditions is in sharp contrast to what children as young as 2 years of age (who also have vocabularies of on average 50–500 words, Fenson et al. 1994) do might be related to that difference in how pointing and words work for word-trained dogs versus children. For example, in a recent series of similar studies, Grassmann and Tomasello (2010) demonstrated that young children integrate pointing and object labels in a different manner than the dogs in the current study did. In particular, the children relied on the pointing gesture when pointing and naming conflicted. Thus, they accepted new exemplars for known words (Familiar Label Conflict) and second labels for known objects (Novel Label Conflict). However, this difference in behavior does not mean that the word-trained dogs fail to understand the communicative intention of the speaker. One must not overlook an important difference in experience with pointing and labeling between the word-trained dogs from the current study and children. As stated above, for children, pointing and words go together from the beginning of referential communication (Carpenter et al. 1998; Gliga and Csibra 2009), and mothers in Western cultures often name objects they point at for their children (Masur 1997). The owners of the dogs in our study report that they do not use pointing in their word training and this might explain the different responses found for children (Grassmann and Tomasello 2010) and the dogs in the current study. Interestingly, Pilley and Reid (2011) report that they used pointing when introducing new labels for Chaser. Thus, Chaser’s experience with pointing and naming might be more similar to children’s experience, and thus, Chaser might be more likely to fetch the pointed-to object in the conflict conditions. In addition, since Chaser has one-to-many mappings in his vocabulary, he might be less “strict” in choosing the labeled object in the conflict conditions.

The current study is thus a first step toward an understanding of how nature and nurture interact in the development of an understanding of reference and communicative intentions in dogs. Future research needs to investigate how pet dogs in general (not only the highly trained dogs) integrate verbal and gestural information in order to make more general conclusions about how dogs integrate these two modalities of human communication.

## Footnotes

1 We thank an anonymous reviewer of an earlier version of this manuscript for suggesting this manipulation.

2 For example, in the Novel Label Baseline, Paddy got 7 trials with a chance probability of 0.2 (object selection out of 5 available objects) and 5 trials with a chance probability of 0.25 (object selection out of 4 available objects).

3 We thank an anonymous reviewer for pointing out this possibility.
